# Baltimore Infectious Diseases Ordinance

**Published:** 1883-01

**Authors:** 


Baltimore Infectious Diseases Ordinance.—This "ordinance to protect the public health" is one that well may serve as a model to Baltimore's sister cities. Certainly no subject is of greater interest to all classes of the people than their own health and how to preserve it from the attacks of dreaded "fevers." Should the ordinance, which contains twenty sections and a large amount of matter, be carried out even in part, the spread of diphtheria, small-pox, cholera, yellow fever and scarlet fever will be prevented, or assuredly limited to very narrow regions, if strict quarantine can check epidemics. But physicians know that to "fence in" a man sick with small-pox or cholera at a season of the year when few or no cases prevail is a very different thing from checking an epidemic by putting sentinels about houses whose inmates have succumbed to atmospheric or tellurial influences, impalpable poisons whose nature, even with the present great advance of pathology, is yet conjectural. We are inclined to regard checking of epidemics, especially those that prevail in the region of our country where this ordinance is issued, as a matter wherein the temperature and atmosphere play a most important part; why else do we pray for frost when the deadly yellow fever is prevailing? True, one disease, namely, variola, has a means of prophylaxis that makes strict police surveillance of great avail, and the ordinance of the Baltimoreans covers all possible emergencies. Yet the fine for refusing to be vaccinated is not to be more than ten dollars. Why, after all the details of guarding against epidemics, the refusal to yield in what nearly every intelligent physician believes a necessity is to be visited by a very mild punishment, is a matter that we cannot clearly understand, unless it be that the municipal government believes all the citizens obedient and intelligent enough to follow physicians' teachings, which we trust is the case.

